# ENIGMA: an enterotype-like unigram mixture model for microbial association analysis

**DOI:** 10.1186/s12864-019-5476-9

**Published:** 2019-04-04

**Authors:** Ko Abe, Masaaki Hirayama, Kinji Ohno, Teppei Shimamura

**Affiliations:** 1 Division of Systems Biology, Nagoya University Graduate School of Medicine, 65 Tsurumai-Cho, Showa-Ku, Nagoya, 466-8550 Japan; 2 School of Health Sciences, Nagoya University Graduate School of Medicine, 1-1-20 Daiko-Minami, Higashi-Ku, Nagoya, 461-8873 Japan; 3 Division of Neurogenetics, Center for Neurological Diseases and Cancer, Nagoya University Graduate School of Medicine, 65 Tsurumai-Cho, Showa-Ku, Nagoya, 466-8550 Japan; 4 Division of Systems Biology, Nagoya University Graduate School of Medicine, 65 Tsurumai-Cho, Showa-Ku, Nagoya, 466-8550 Japan

**Keywords:** Enterotype, Topic model, Unigram mixture, Bayesian inference, Metagenomics

## Abstract

**Background:**

One of the major challenges in microbial studies is detecting associations between microbial communities and a specific disease. A specialized feature of microbiome count data is that intestinal bacterial communities form clusters called as “enterotype”, which are characterized by differences in specific bacterial taxa, making it difficult to analyze these data under health and disease conditions. Traditional probabilistic modeling cannot distinguish between the bacterial differences derived from enterotype and those related to a specific disease.

**Results:**

We propose a new probabilistic model, named as ENIGMA (Enterotype-like uNIGram mixture model for Microbial Association analysis), which can be used to address these problems. ENIGMA enabled simultaneous estimation of enterotype-like clusters characterized by the abundances of signature bacterial genera and the parameters of environmental effects associated with the disease.

**Conclusion:**

In the simulation study, we evaluated the accuracy of parameter estimation. Furthermore, by analyzing the real-world data, we detected the bacteria related to Parkinson’s disease. ENIGMA is implemented in R and is available from GitHub (https://github.com/abikoushi/enigma).

## Background

More than 100 trillion microbes live on and within human beings and form of complex microbial communities (microbiota). Most microbes cannot be cultured in laboratories, making it difficult to understand how individual microorganisms mediate vital microbiome-host interactions under health and disease conditions. However, recent important advances in high-throughput sequencing technology have enabled observation of the composition of these intestinal microbes. For each sample drawn from an ecosystem, the number of occurrences of each operational taxonomic units (OTUs) is measured and the resulting OTU abundance can be summarized at any level of the bacterial phylogeny. Discovering recurrent microbial compositional patterns that are related to a specific disease is a significant challenge, as individuals with the same disease typically harbor different microbial community structures.

Recent large-scale sequencing surveys of the human intestinal microbiome, such as the US NIH Human Microbiome Project (HMP) and the European Metagenomics of the Human Intestinal Tract project (MetaHIT), have revealed considerable variations in microbiota composition among individuals [1, 2]. Particularly, community clusters characterized by differences in the abundance of signature taxa, referred to as enterotypes, were first reported in humans [3]. Later, other studies identified enterotype-like clusters that may reflect features of the host-microbial physiology and homeostasis in different species [4, 5] or at different human body sites [6–9]. This microbial stratification has motivated the development of methods for examining unknown clusters of microbial communities.

Probabilistic modeling of microbial metagenomics data often provides a powerful framework for characterizing the microbial community structures [10–12]. For example, Knights et al. [10] applied a Dirichlet prior to a single-level hierarchy and proposed a Bayesian approach for estimating the proportion of microbial communities. Holmes et al. [11] extended the Dirichlet prior to Dirichlet multinomial mixtures to facilitate clustering of microbiome samples. Shafiei et al. [12] proposed a hierarchical model for Bayesian inference of microbial communities (BioMiCo) to identify clusters of OTUs related to environmental factors of interest.

However, such models are not suitable for discovering enterotype-like clusters of microbial communities and associations between microbes and a specific disease for the following two reasons. First, the frameworks of Knights et al. [10] and Holmes et al. [11] do not explicitly address the association between the microbial compositional patterns and environmental depend on the interest. Second, the framework of Shafiei et al. [12] models the structure of each sample using a hierarchical mixture of multinomial distributions that are depends on the factors of interest. Individual host properties such as body mass index, age, or gender cannot explain the observed enterotypes [3]. Thus, such enterotype-like clusters that describes interindividual variability among humans do not always to directly affect host probabilities such as diseases ranging from localized gastroenterologic disorders to neurologic, respiratory, metabolic hepatic, and cardiovascular illnesses.

Here, we introduce a novel probabilistic model of a microbial community structures, named as ENIGMA (Enterotype-like uNIGram mixture model for Microbial Association analysis), to address these problems. ENIGMA includes the following contributions:

1. ENIGMA uses OTU abundances as input and models each sample by the underlying unigram mixture whose parameters are represented by unknown group effects and known effects of interest. The group effects are represented by baseline parameters that change with a latent group of microbial communities. One of the most important features of our model is that the group effects are independent of the effects of interest. This enables the separation of interindividual variability and fixed effects of the host properties related to disease risk.
2. ENIGMA is regarded as Bayesian learning for detecting associations between a community structure and factors of interest. Our model can be used to simultaneously learn how enterotype-like clusters of OTUs contribute to the microbial structure and how microbial compositional patterns may be related to known features of the sample.
3. We provide an efficient learning procedure for ENIGMA by using a Laplace approximation to integrate latent variables and estimate the evidence of the complete model and credible intervals of the parameters. The software package that implements ENIGMA in the R environment is available from https://github.com/abikoushi/enigma.

We describe our proposed framework and algorithm in the “Methods” section. We evaluate the performance of ENIGMA using simulated data in terms of its accuracy to estimate parameters and identify clusters in the “Simulation study” section. We apply ENIGMA to clinical metagenomics data and demonstrate how ENIGMA simultaneously identifies enterotype-like clusters and gut microbiota related to Parkinson’s disease (PD) in the “Results on real data” section.

## Methods

**Fig. 1:**
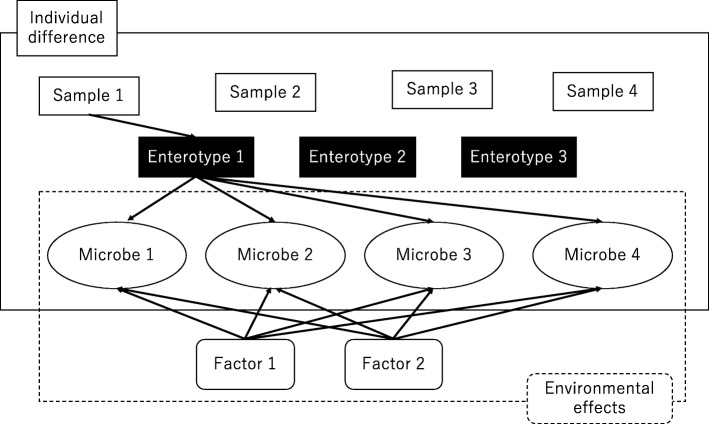
A conceptual view of ENIGMA

The key idea of ENIGMA is to adjust for the effects of the enterotype and evaluate the increases and decreases of bacterial abundance associated with environmental factors. Figure 1 shows a conceptual view of ENIGMA. The formal definition of the model is described in the following Mode section. Here we introduce several notations.

Suppose that we observe microbiome count data of *K* taxa for *N* samples with *M* individual host properties, (*y*_*nk*_,*x*_*nm*_) (*n*=1,…,*n*;*k*=1,…,*K*;*m*=1,…,*M*) where $y_{nk}\in \mathbb {N}$ represents the abundance of the *k*-th taxa in the *n*-th sample and *x*_*nm*_ represents a binary variable such that *x*_*nm*_=1 if the *n*-th sample has the *m*-th host property and is otherwise *x*_*nm*_=0. Here the word taxa can represent any level of the bacterial phylogeny, e.g., species, genes, family, order, etc.

### Model

**Fig. 2:**
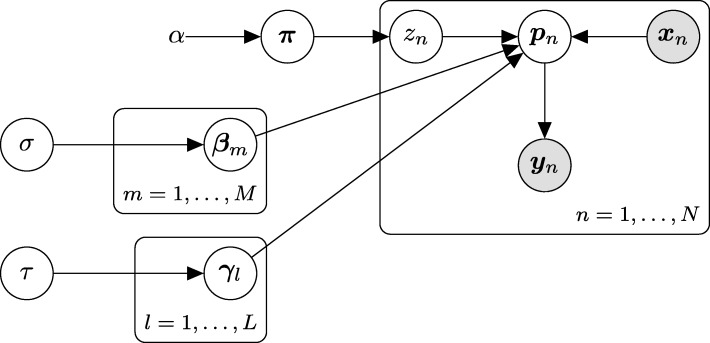
Plate diagram of the model for ENIGMA ***y***_*n*_ is affected from environmental factors ***x***_*n*_ and latent variables ***z***_*n*_

Figure 2 shows a plate diagram of the proposed model for metagenome sequencing, where ***y***_*n*_ is the read count vector of the *n*-th sample, ***x***_*n*_ is the vector of the host properties of the *n*-th sample and *z*_*n*_∈{1,…,*L*} is a latent class of the *n*-th sample. Our model is a simple extension of the unigram mixture model. We assume that each sample is generated from a multinomial distribution with the parameter vector ***p***_*n*_=(*p*_*n*1_,…,*p*_*nK*_)^⊤^. The elements of ***p***_*n*_ and *p*_*nk*_ (*k*=1,…,*K*) are probabilities of the occurrence of the *K* taxa for the *n*-th sample. We also assume that *p*_*nk*_ can be influenced independently by the environmental factor on the taxa that is common to all latent classes and the interindividual factor on the latent enterotype-like classes. More specifically, the generative process of ENIGMA is defined as follows:

1 $$\begin{array}{*{20}l} \boldsymbol{y}_{n}|z_{n},x_{n},\boldsymbol{\beta} &\sim \text{Multinomial}\left(\boldsymbol{p}_{n}\right)  \\ \boldsymbol{p}_{n} &= \text{softmax}\left(\boldsymbol{\gamma}_{z_{n}} + \boldsymbol{x}_{n}\boldsymbol{B}\right) \\ z_{n}|\boldsymbol{\pi} &\sim \text{Categorical}(\boldsymbol{\pi}) \\ \boldsymbol{\pi}|\boldsymbol{\alpha} &\sim \text{Dirichlet}(\boldsymbol{\alpha})  \\ \boldsymbol{\beta}_{m} &\sim \text{Normal}_{K}\left(O_{K},\sigma^{2} I_{K}\right)  \\ \boldsymbol{\gamma}_{l} &\sim \text{Normal}_{K}\left(O_{K},\tau^{2} I_{K}\right)  \end{array} $$

where ***γ***_*l*_ is baseline parameter (*K*-dimensional vector) that changes with the latent class, *M*×*K* matrix $\boldsymbol {B}=\left (\beta _{mk}\right)$ is effect of a environmental factor common to all enterotype-like clusters, ***β***_*m*_ is a *m*-th row-vector of ***B***, $\boldsymbol {\pi }=\left (\pi _{1},\ldots,\pi _{L}\right)$ is a mixing ratio of components, *O*_*K*_ is a *K*-dimensional zero matrix and *I*_*K*_ is *K*-dimensional identity matrix. Here, the softmax function is defined by $\text {softmax}(\boldsymbol {x})=\frac {\exp (\boldsymbol {x})}{\sum _{k=1}^{K}{\exp (x_{k})}}$ for a vector ***x***=(*x*_1_,…,*x*_*K*_)^⊤^ using an element-wise exponential function and the probability function of categorical distribution is parameterized as $\Pr (z=l |\boldsymbol {\pi }) = \pi _{l}$, *l*∈{1,…,*L*}. In a Bayesian approach, the prior distributions for ***π***, ***β***, and ***γ***_*l*_ must be defiend. We set a prior based on the Dirichlet distribution for ***π***, and flat priors to the hyperparameters *σ* and *τ* for ***β*** and ***γ***, respectively. For the convenience of later section, let ***p****l*′=softmax(***γ***_*l*_) be the probabilities of the occurrence of bacteria in the latent classes *l*.

### Parameter estimation

Let us denote observed matrix by ***Y***=(*y*_*nk*_), ***X***=(*x*_*nm*_), the unknown parameters by ***θ***=(***α***,***B***,***γ***_1_,…,***γ***_*L*_,*σ*,*τ*), and their prior by *ϕ*(***θ***). The posterior distribution is represented as follows:

2 $$\begin{array}{*{20}l} p(\boldsymbol{\theta}, \boldsymbol{z} |\boldsymbol{Y}) \propto \prod_{n=1}^{N} p(\boldsymbol{y}_{n}|z_{n},\boldsymbol{x}_{n},\boldsymbol{\theta})p(z_{n}|\boldsymbol{\theta})\phi(\boldsymbol{\theta}) \end{array} $$

First, latent variable *z*_*n*_ must be marginalized. The likelihood is described by

3 $$ \prod_{n=1}^{N} p(\boldsymbol{y}_{n}|\boldsymbol{x}_{n},\boldsymbol{\theta}) = \prod_{n=1}^{N}\sum_{l=1}^{L}\pi_{l} p(\boldsymbol{y}_{n}|z_{n}=l,\boldsymbol{x}_{n},\boldsymbol{\theta}).  $$

The posterior distribution is proportional to the product of the likelihood and prior density:

$$\begin{array}{*{20}l} p(\boldsymbol{\theta}|\boldsymbol{Y}) &\propto \exp\left\{\sum_{n=1}^{N} \log p\left(\boldsymbol{y}_{n}|\boldsymbol{x}_{n},\boldsymbol{\theta}\right) + \log \phi (\boldsymbol{\theta}) \right\} \end{array} $$

Let $\boldsymbol {\hat \theta }$ be the MAP estimator of ***θ***, found by maximizing $\log p(\boldsymbol {\theta },\boldsymbol {Y},\boldsymbol {X})$.

We use a Laplace approximation [13] for parameter estimation. A Taylor expansion around $\boldsymbol {\hat \theta }$ gives

4 $$\begin{array}{*{20}l} \log p(\boldsymbol{\theta} | \boldsymbol{Y}, \boldsymbol{X}) \approx \log p(\boldsymbol{\hat \theta}| \boldsymbol{Y}, \boldsymbol{X}) + \frac{1}{2}(\boldsymbol{\theta} - \boldsymbol{\hat \theta})^{\top} H(\boldsymbol{\hat \theta})(\boldsymbol{\theta} - \boldsymbol{\hat \theta})  \end{array} $$

where $H(\boldsymbol {\hat \theta })$ is the Hessian of $\log p(\boldsymbol {\theta } | \boldsymbol {Y}, \boldsymbol {X})$ evaluated at $\boldsymbol {\hat \theta }$. Eq. 4 gives

$$\begin{array}{*{20}l} p(\boldsymbol{\theta} | \boldsymbol{Y}, \boldsymbol{X}) \approx \frac{1}{C} \exp\left\{\frac{1}{2}(\boldsymbol{\theta} - \boldsymbol{\hat \theta})^{\top} H\left(\boldsymbol{\hat \theta}\right)\left(\boldsymbol{\theta} - \boldsymbol{\hat \theta}\right) \right\} \end{array} $$

where *C* is a normalizing constant. This relationship shows that *p*(*θ*|***Y***,***X***) can be approximated by the normal distribution $N\left (\boldsymbol {\hat \theta }, H^{-1}\left (\boldsymbol {\hat \theta }\right)\right)$. Credible intervals can be calculated from this multivariate normal distribution.

We used the stochastic programming language Stan (http://mc-stan.org/) for its implementation. The MAP estimators were obtained by the L-BFGS method. Credible intervals were computed from the using a Stan function to compute the Hessian at the MAP estimates.

After fitting the model, the enterotype-like cluster of each sample must be classified. The conditional probability of *z*_*n*_=*l* is

5 $$ \Pr(z_{n}=l)=\frac{\pi_{l} p(\boldsymbol{y}_{n}|\boldsymbol{\gamma}_{l}, \boldsymbol{\beta}, \boldsymbol{x}_{n})}{\sum_{l=1}^{L}\pi_{l} p(\boldsymbol{y}_{n}|\boldsymbol{\gamma}_{l},\boldsymbol{\beta}, \boldsymbol{x}_{n})}.   $$

This is the probability that the *n*-th sample belongs to cluster *l*. Next, the *n*-th sample is then classified into the *l*-th cluster that maximizes the conditional probability given by Eq. 5.

### Model Selection

We also examined whether or not the whole set rather than individual bacteria is related to the environmental factors of interest. We compared between the two models when ***B***≠***0*** and ***B***=***0***. We used the log marginal likelihood as the goodness of fit for model comparison. The marginal likelihood is given by

6 $$\begin{array}{*{20}l} P(\boldsymbol{Y}|\boldsymbol{X}) = \int p(\boldsymbol{Y},\boldsymbol{\theta}|\boldsymbol{X}) \, d\boldsymbol{\theta}. \end{array} $$

From Eq. 4, we have

7 $$ {}{\begin{aligned} \int\! p(\boldsymbol{\theta}, \boldsymbol{Y}|\boldsymbol{X}) \, d \boldsymbol{\theta} \approx p\left(\boldsymbol{\hat \theta}| \boldsymbol{Y}, \boldsymbol{X}\right) \int\! \exp\left(\frac{1}{2}\left(\boldsymbol{\theta} - \boldsymbol{\hat \theta}\right)^{\top} H\left(\boldsymbol{\hat \theta}\right)\left(\boldsymbol{\theta} - \boldsymbol{\hat \theta}\right)\right) \, d \, \boldsymbol{\theta}. \end{aligned}}  $$

Thus, the log marginal likelihood is approximated by the following formula:

8 $$ {}{\begin{aligned} \log P(\boldsymbol{Y}|\boldsymbol{X}) \approx \log p\left(\boldsymbol{Y} | \boldsymbol{\hat \theta}, \boldsymbol{X}\right) + \phi\left(\boldsymbol{\hat \theta}\right) + \frac{D}{2}\log{2 \pi} - \frac{1}{2} \log |H\left(\boldsymbol{\hat \theta}\right)|  \end{aligned}}  $$

where *D* is the number of free parameters. In model comparison, we choose the model showing larger log marginal likelihood.

## Simulation study

To demonstrate the performance of ENIGMA, we conducted several simulation experiments. The synthetic data were naturally produced via our generative process given by Eq. 1. We set *M*=2000, *L*=3, *π*_*l*_=1/3, and ***α***=(1,1,1)^*T*^. We first generated ***B*** and ***γ***_*l*_ from the standard normal distribution. The variables ***x***_*n*_, *z*_*n*_, and ***y***_*n*_ are then sampled from the Bernoulli distribution with probability of 0.5, the categorical distribution, and the multinomial distribution, respectively. For the above parameter settings, we randomly generated a count dataset of 100 taxa for 100 samples for evaluation.

- **Coverage probability (CP)**: The coverage probability is the proportion of the time over which the interval contains the true value. A discrepancy between the coverage probability and the nominal coverage probability frequently occurs. When the actual coverage is greater than the nominal coverage, the interval is referred to as conservative. If the interval is conservative, there is no inconsistency in interpretation.
- **Bias**: The bias of ***B*** is defined by the difference between true value and estimated value $E[\hat {\boldsymbol {B}}]-\boldsymbol {B}$.
- **Standard error (SE)**: The standard error is the standard deviation from the estimate. A smaller standard error indicates the higher accuracy of estimation.
- **Root mean squared error (RMSE)**: The RMSE is defined by
  $\sqrt {E[\left (\boldsymbol {\hat {B}} - \mathbf {B}\right)^{2}]}$. A smaller RMSE indicates the higher accuracy of the estimation.
- **Accuracy**: The accuracy is the percentage of samples correctly classified into original group.

To calcurate these metrics, we detrmined that we calculated the sample means and standard deviations of $\hat {\boldsymbol {B}}$ and $\left (\hat {\boldsymbol {B}}-\boldsymbol {B}\right)^{2}$ from the 10,000 synthetic datasets.

**Fig. 3:**
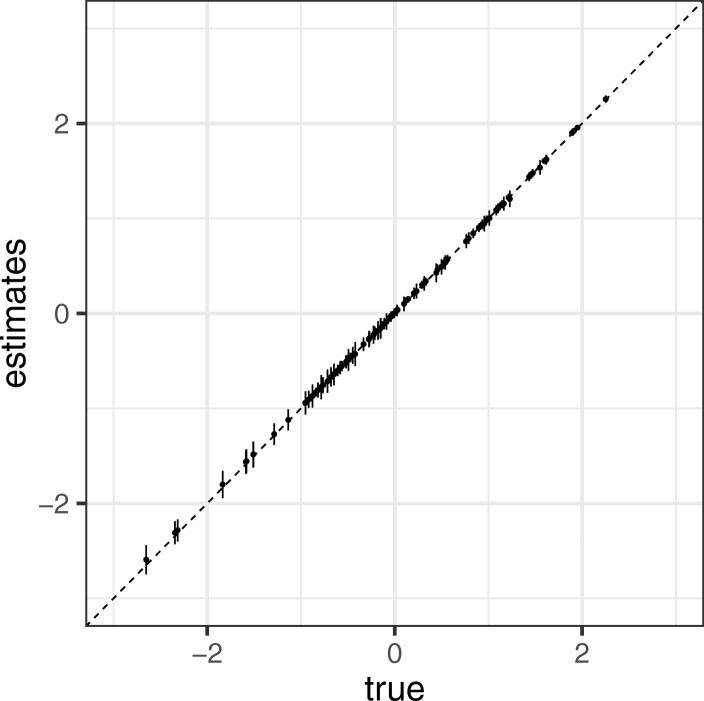
Simulation result of ***B*** The comparison true ***B*** and the mean of $\boldsymbol {\hat {B}}$. The error bars indicates SE

**Fig. 4:**
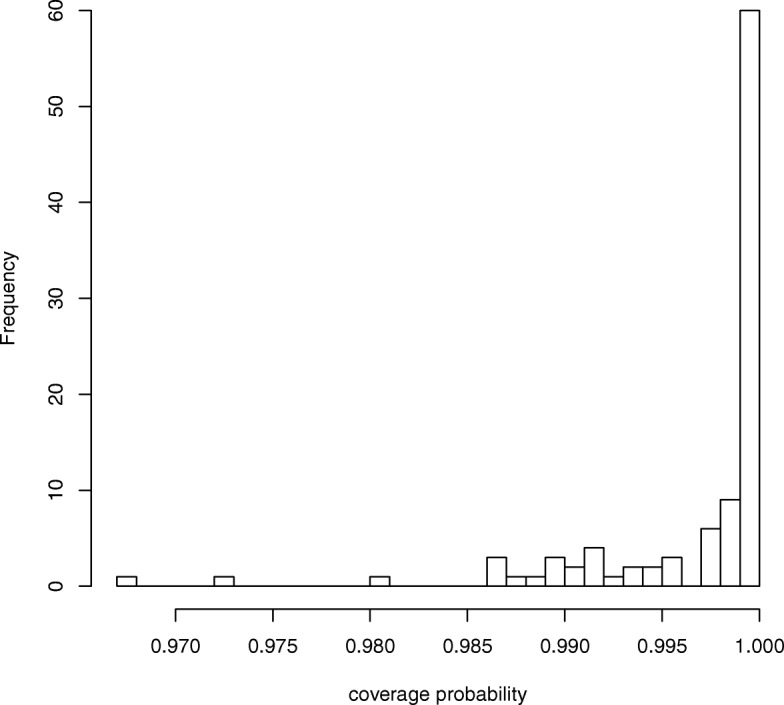
Coverage probability of ***B***. The histogram of coverage probability of ***B***

Figure 3 shows a comparison of the true ***B*** and the mean and standard deviation of estimates $\hat {\boldsymbol {B}}$ obtained from the 10,000 simulations. We observed that the points were arranged diagonally, indicating that the estimator of ENIGMA was unbiased. We also calculated the proportion of the time for which the 95% credible interval contains the true value of ***B***. We found that this proportion was greater than nominal value of 0.95 for all ***B*** in Fig. 4. Table 1 shows the coverage probability (CP), bias, standard error (SE), and RMSE of $\hat {\boldsymbol {B}}$, respectively. We observed that the bias and standard error decreased when *β*_*mk*_ was large (i.e. the corresponding abundance was large). We also found that the accuracy of classification given by Eq. 5 was exactly 100%. Thus, these results indicate that ENIGMA can produce reasonable estimates.

**Table 1:** Coverage probability (CP), bias, standard error (SE), and RMSE of $\boldsymbol {\hat {B}}$

***β***	CP	Bias	SE	RMSE	***β***	CP	Bias	SE	RMSE
-3.40	0.97	0.08	0.15	0.17	-0.04	1.00	0.01	0.05	0.05
-2.65	0.97	0.06	0.15	0.16	-0.04	1.00	0.01	0.05	0.05
-2.34	0.99	0.04	0.12	0.13	-0.01	1.00	0.01	0.05	0.05
-2.32	0.99	0.03	0.12	0.12	0.01	1.00	0.01	0.04	0.04
-1.83	0.98	0.03	0.14	0.15	0.02	1.00	0.01	0.06	0.06
-1.59	0.99	0.02	0.13	0.13	0.02	1.00	0.01	0.04	0.05
-1.58	0.99	0.03	0.13	0.13	0.03	1.00	0.01	0.04	0.04
-1.51	0.99	0.02	0.14	0.14	0.10	1.00	-0.00	0.08	0.08
-1.51	0.99	0.02	0.13	0.13	0.13	1.00	0.01	0.03	0.03
-1.29	0.99	0.02	0.11	0.11	0.14	1.00	0.01	0.03	0.03
-1.14	0.99	0.01	0.11	0.11	0.21	1.00	0.01	0.06	0.06
-0.95	1.00	0.01	0.09	0.09	0.23	1.00	0.00	0.08	0.08
-0.95	0.99	0.01	0.12	0.12	0.29	1.00	0.01	0.04	0.04
-0.92	1.00	0.01	0.09	0.09	0.31	1.00	0.01	0.05	0.05
-0.88	0.99	0.01	0.12	0.12	0.32	1.00	0.00	0.08	0.08
-0.84	1.00	0.01	0.05	0.05	0.33	1.00	0.01	0.04	0.04
-0.82	1.00	0.01	0.08	0.08	0.44	0.99	-0.02	0.10	0.10
-0.78	0.99	0.01	0.13	0.13	0.46	1.00	0.01	0.05	0.05
-0.78	1.00	0.01	0.07	0.07	0.50	1.00	-0.01	0.08	0.08
-0.76	1.00	0.01	0.08	0.08	0.53	1.00	0.00	0.06	0.06
-0.72	0.99	0.00	0.12	0.12	0.54	1.00	-0.00	0.08	0.08
-0.68	1.00	0.01	0.10	0.10	0.55	1.00	0.01	0.04	0.04
-0.65	0.99	0.01	0.11	0.11	0.55	1.00	0.01	0.03	0.03
-0.65	0.99	0.01	0.11	0.11	0.56	1.00	0.01	0.05	0.05
-0.65	1.00	0.01	0.06	0.06	0.76	1.00	-0.00	0.07	0.07
-0.61	1.00	0.01	0.06	0.06	0.79	1.00	0.00	0.06	0.06
-0.58	1.00	0.01	0.06	0.06	0.84	1.00	0.00	0.05	0.05
-0.58	1.00	0.01	0.07	0.07	0.90	1.00	0.01	0.04	0.04
-0.56	1.00	0.01	0.05	0.05	0.93	1.00	0.00	0.05	0.05
-0.52	1.00	0.01	0.06	0.06	0.96	1.00	-0.01	0.08	0.08
-0.52	1.00	0.01	0.07	0.07	0.98	1.00	0.01	0.04	0.04
-0.51	1.00	0.01	0.04	0.05	1.01	1.00	-0.01	0.08	0.08
-0.50	1.00	0.01	0.05	0.05	1.08	1.00	0.00	0.05	0.06
-0.50	1.00	0.01	0.04	0.04	1.10	1.00	0.00	0.05	0.05
-0.49	0.99	0.00	0.11	0.11	1.13	1.00	0.01	0.04	0.04
-0.47	1.00	0.01	0.05	0.05	1.14	1.00	0.01	0.04	0.04
-0.45	1.00	0.01	0.09	0.09	1.16	1.00	-0.01	0.07	0.07
-0.42	0.99	-0.01	0.13	0.13	1.22	1.00	0.01	0.04	0.04
-0.33	1.00	0.01	0.07	0.07	1.23	1.00	-0.02	0.09	0.09
-0.28	1.00	0.00	0.09	0.09	1.43	1.00	0.00	0.04	0.04
-0.27	1.00	0.01	0.07	0.07	1.45	1.00	0.01	0.04	0.04
-0.23	1.00	0.00	0.09	0.09	1.47	1.00	0.00	0.04	0.04
-0.21	1.00	0.01	0.07	0.07	1.55	1.00	-0.01	0.07	0.08
-0.18	1.00	0.00	0.10	0.10	1.60	1.00	0.01	0.03	0.03
-0.15	0.99	-0.01	0.11	0.11	1.61	1.00	0.00	0.05	0.05
-0.11	1.00	0.01	0.06	0.06	1.89	1.00	0.01	0.03	0.03
-0.09	1.00	0.00	0.09	0.09	1.91	1.00	0.01	0.03	0.03
-0.05	1.00	0.01	0.04	0.04	1.95	1.00	0.01	0.02	0.02
-0.05	1.00	0.01	0.04	0.04	2.25	1.00	0.00	0.04	0.04

## Results on real data

### Arumugam et al. (2011)’s data

We demonstrated that the enterotype-like cluster can be estimated using the data of Arumugam et al. [3]. This data is *N*=33, *K*=55. The data of Arumugam et al. [3] does not disclose the total read count. Thus, We used the relative abundance multiplied by 10,000 as *y*_*nk*_. Based on the result of Arumugam et al. [3], the number of latent classes in ENIGMA was chosen to be *L*=3. We estimated the parameters using the ENIGMA and setting all *β*_*mk*_=0 in Eq. 1. We set the hyperparameters of Dirichlet prior ***α***=(1,…,1)^⊤^, which is equivalent to a noninformative prior.

**Fig. 5:**
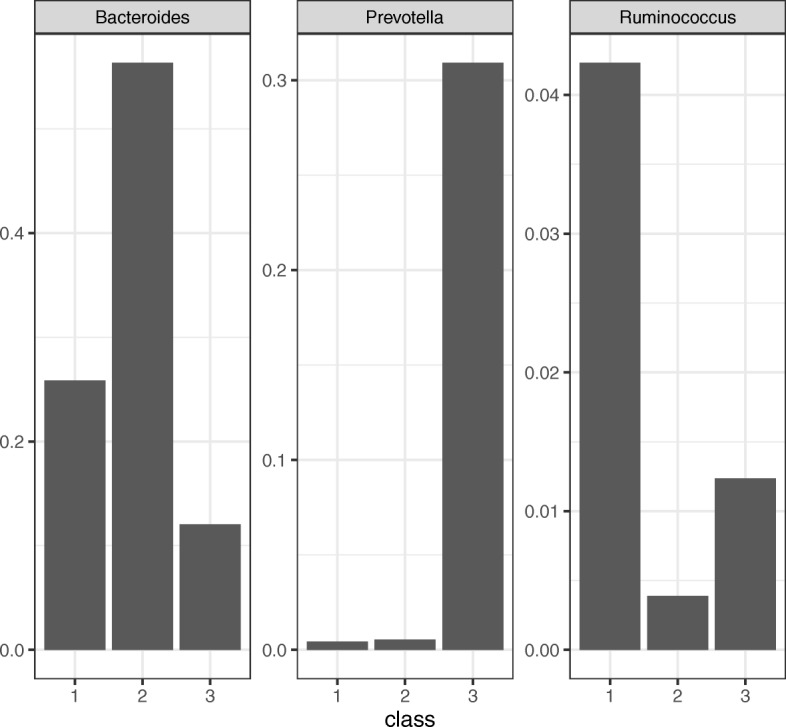
Probability of occurrence in three bacteria

Arumugam et al. [3] showed that the enterotype is characterized by the differences in the abundance of *Bacteroides*, *Prevotella*, and *Ruminococcus*. Estimates of the probability of occurrence of those bacteria in three clusters are shown in the Fig. 5. Class 1 contains high-level *Ruminococcus*, class 2 contains high-level *Bacteroides*, and class 3 contains high-level *Prevotella*. This result is consistent with that of Arumugam et al. (2011) [3].

### Parkinson’s disease data

To validate the performance of ENIGMA in discovering clusters of microbial communities and associations between microbes and a specific disease, we applied ENIGMA to the real metagenomic sequencing data from Scheperjans et al. [14], Hill-Burns et al. [15], Heintz-Buschart et al. [16] and Hopfner et al. [17]. The data was analyzed by sequencing the bacterial 16S ribosomal RNA genes sampled from patients with Parkinson’s disease (PD) and controls in Finland, USA, and Germany. Table 2 shows the summary statistics of the data. The OTUs were mapped to the SILVA taxonomic reference, version 132 (https://www.arb-silva.de/) and the abundances of family-level taxa were calculated.

**Table 2:** Data summary

	PD	CO
Finland	74	74
German	55	64
USA	207	139

To assess the optimal number of clusters, we used the Calinski-Harabasz (CH) Index. It is defined as:

9 $$\begin{array}{*{20}l} \text{CH}_{l} = \frac{\text{BC}_{l}/(l-1)}{\text{WC}_{l}/(n-l)} \end{array} $$

where BC_*l*_ is the between-cluster sum of squares (i.e. the squared distances between all points *i* and *j*, for which *i* and *j* are not in the same cluster) and WC_*l*_ is the within-clusters sum of squares (i.e. the squared distances between all points *i* and *j*, for which *i* and *j* are in the same cluster). Here, we used Jensen-Shannon divergence (JSD) as the distance. The JSD between samples ***a***=(*a*_1_,…,*a*_*K*_) and ***b***=(*b*_1_,…,*b*_*K*_) is defined as follows:

10 $$\begin{array}{*{20}l} \text{JSD}(\boldsymbol{a},\boldsymbol{b}) = \frac{1}{2} \left(\sum_{k=1}^{K} a_{k} \log(a_{k}/b_{k})) + \sum_{k=1}^{K} b_{k} \log(b_{k}/a_{k})) \right). \end{array} $$

**Fig. 6:**
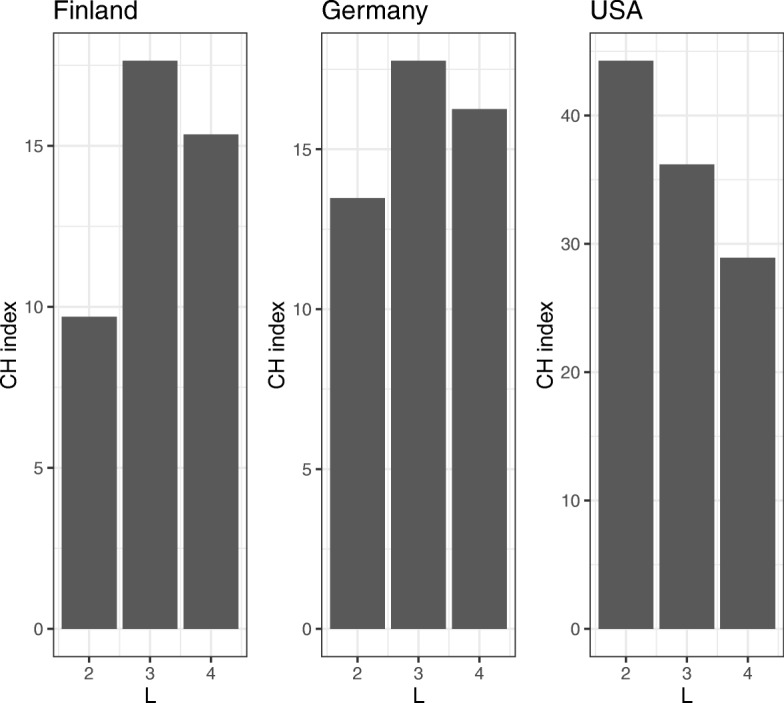
The CH indexes in each country

When calculating the JSD, we used the normalized abundance obtained by dividing *y*_*nk*_ by the total read count, and 0 was replaced with pseudo count 10^−6^. We chose the number of clusters *L* such that CH_*l*_ was maximal. To evaluate the CH Index, we use the function index.G1() from the R library clusterSim. The number of latent classes in ENIGMA was chosen to be *L*=3 in Finland and Germany and *L*=2 in USA by the CH indexes (Fig. 6).

First, we evaluated whether the model assumption was satisfied when using this data. According to Arumugam et al. (2011) [3], the gender of the host is not related with the enterotype. The genders of the subjects were published in the Finland study. We examined the relationship between gender and enterotype-like cluster using the data from Finland. Table 3 shows there was no correlation between them. We conducted a Chi-squared test for independence as shown in Table 3 and the *p*-value was 0.66.

**Table 3:** Cross-tabulation of gender and cluster

Class	1	2	3
Female	22	31	21
Male	21	27	26

We evaluated whether the model showing that bacteria were associated with PD is better than the model without the associations in terms of marginal likelihood. Marginal likelihood represents the model evidence expressing the preference of the data for different models. Let $\mathcal {M}_{1}$ be the model which is described by Eq. 1 and $\mathcal {M}_{0}$ be the model setting all *β*_*mk*_=0 in Eq. 1. Table 4 shows that the marginal likelihood of $\mathcal {M}_{1}$ was greater than $\mathcal {M}_{0}$. It is preferrd to explain the data by considering the association between the microbiota and PD.

**Table 4:** Comparison marginal likelihood

	Finland	Germany	USA
$\mathcal {M}_{0}$	-442734.62	-5913441.14	-3010279.35
$\mathcal {M}_{1}$	-355079.50	-3807297.76	-2063932.02

**Fig. 7:**
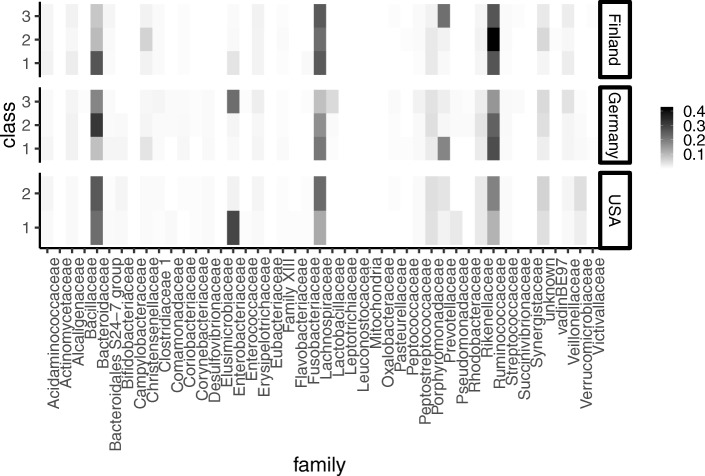
Heatmap showing $\left (\boldsymbol {\hat p}_l'\right).$ These quantities correspond to the probabilities of the occurrences of bacteria for the three latent classes

**Fig. 8:**
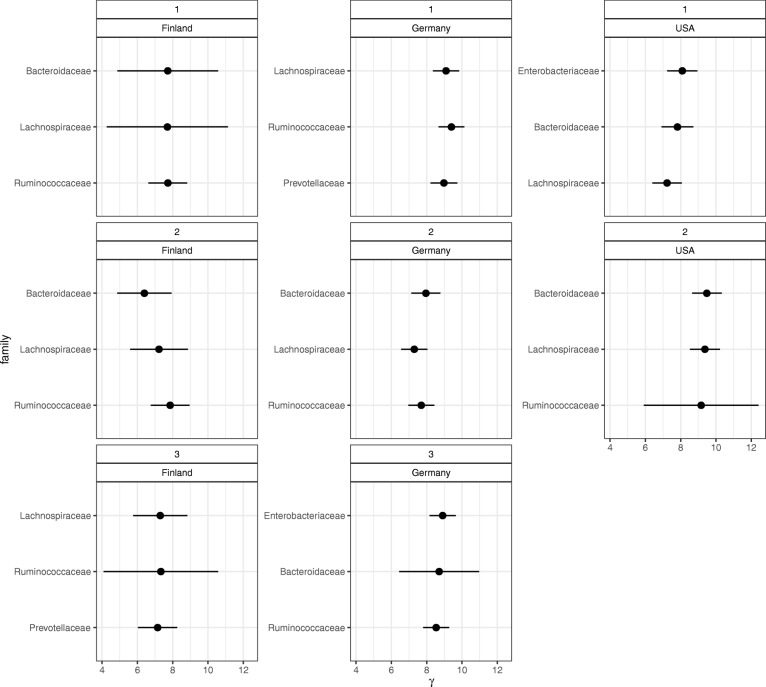
Top three $\left (\boldsymbol {\hat \gamma }_{l}\right)'$ in each country

Figure 7 shows the estimated probabilities of the occurrences of bacteria for the three latent classes, ***p****l*′, (*l*=1,2,3). Bacteria detected in fewer three countries were removed. Arumugam et al. [3] showed that enterotype is characterized by the differences in the abundance of *Bacteroides*, *Prevotella*, and *Ruminococcus*. The results of ENIGMA showed the same tendency as the previous survey. Figure 8 shows the $(\boldsymbol {\hat \gamma }_l)'$ values and their credible intervals. The top three microbes in each enterotype-like cluster are shown in excerpts for this plot. According to the results of ENIGMA, the abundance of *Enterobacteriaceae* and *Lachnospiraceae* also differed greatly among clusters. Bacterial abundance differed between countries. In the USA, there was a high abundance of *Verrucomicrobiaceae*, while in Finland, few of these bacteria were detected. In contrast, Finland showed more *Prevotellaceae*, with fewer in the in USA it is less.

Table 5 shows the coefficients whose 95% credible intervals did not contain zero in more than two countries. The microbes with these coefficients indicates that the corresponding microbial composition patterns were significantly related to PD. We found that at the family levels, *Clostridiaceae*, *Comamonadaceae*, *Prevotellaceae*, *Actinomycetaceae*, *Bifidobacteriaceae*, *Enterococcaceae*, *Synergistaceae*, *Verrucomicrobiaceae* and *Victivallaceae*, the signs of the coefficients matched in all countries. These results are consistent with those of previous studies. Hill-Burns et al. [15] reported that patients with PD contained high levels of *Bifidobacteriaceae* and *Verrucomicrobiaceae*. Scheperjans et al. [14] reported PD patients contained high levels of *Verrucomicrobiaceae* and low levels of *Prevotellaceae*. Hopfner et al. reported that patients with PD have high levels of *Enterococcaceae*.

**Table 5:** Bacteria significantly associated with PD in more than two countries

	Finland			Germany			USA		
Family	$\hat {\beta }$	Lower bound	Upper bound	$\hat {\beta }$	Lower bound	Upper bound	$\hat {\beta }$	Lower bound	Upper bound
Anaeroplasmataceae	-0.87	-1.28	-0.45	-1.69	-2.03	-1.35	-	-	-
Bacteroidales S24-7 group	-0.52	-0.93	-0.11	0.22	-0.12	0.56	-0.80	-1.16	-0.44
Bradyrhizobiaceae	-	-	-	-0.82	-1.17	-0.47	-1.44	-2.21	-0.66
Brevibacteriaceae	-	-	-	-1.02	-1.38	-0.66	-0.65	-1.05	-0.25
Brucellaceae	-	-	-	-1.69	-2.50	-0.87	-1.34	-1.75	-0.92
Clostridiaceae 1	-0.54	-0.96	-0.13	-0.08	-0.42	0.26	-0.52	-0.88	-0.16
Comamonadaceae	-0.85	-1.35	-0.35	-1.27	-1.61	-0.93	-0.21	-0.57	0.15
Elusimicrobiaceae	-4.17	-5.60	-2.74	-2.11	-2.54	-1.68	2.52	1.03	4.01
Intrasporangiaceae	-	-	-	-3.47	-4.86	-2.07	-3.00	-4.72	-1.28
Leuconostocaceae	-2.66	-4.30	-1.02	0.50	0.13	0.86	-1.74	-2.22	-1.25
Moraxellaceae	-	-	-	-1.58	-1.92	-1.24	-0.92	-1.28	-0.56
Pasteurellaceae	-1.62	-2.07	-1.17	0.30	-0.04	0.64	-1.88	-2.25	-1.51
Prevotellaceae	-2.46	-2.87	-2.05	-0.03	-0.37	0.30	-0.53	-0.89	-0.17
Rhodocyclaceae	-	-	-	-3.53	-4.93	-2.13	-0.75	-1.18	-0.32
Actinomycetaceae	0.11	-0.78	1.01	0.42	0.07	0.78	0.91	0.54	1.28
Bacillaceae	1.72	0.34	3.11	-2.35	-2.72	-1.99	0.80	0.43	1.17
Bdellovibrionaceae	-	-	-	1.43	0.40	2.46	3.07	1.78	4.36
Bifidobacteriaceae	1.34	0.82	1.86	0.54	0.20	0.88	0.01	-0.35	0.37
Campylobacteraceae	0.36	-0.31	1.03	4.90	4.48	5.33	0.83	0.46	1.21
Cytophagaceae	-	-	-	2.45	1.56	3.34	1.70	0.27	3.13
Enterococcaceae	3.87	2.70	5.05	0.74	0.40	1.08	0.09	-0.28	0.45
Lactobacillaceae	3.00	2.56	3.43	-0.51	-0.85	-0.18	1.73	1.36	2.09
Leptotrichiaceae	-0.90	-1.89	0.09	2.57	1.88	3.26	0.82	0.36	1.27
Methanobacteriaceae	-	-	-	0.93	0.59	1.27	0.67	0.30	1.04
Mitochondria	0.60	-1.27	2.46	0.73	0.11	1.36	1.57	0.95	2.20
Paenibacillaceae	-	-	-	2.19	1.28	3.10	1.71	1.30	2.12
Planococcaceae	-	-	-	1.06	0.72	1.41	3.26	2.67	3.85
Rhizobiaceae	-	-	-	0.64	0.24	1.03	1.52	1.08	1.95
Streptococcaceae	0.44	0.03	0.86	0.84	0.50	1.17	0.26	-0.10	0.62
Succinivibrionaceae	-0.32	-0.76	0.11	0.74	0.40	1.08	4.31	3.76	4.86
Synergistaceae	1.26	0.80	1.71	0.25	-0.10	0.61	1.44	1.06	1.82
Verrucomicrobiaceae	1.71	1.23	2.19	1.62	1.29	1.96	-0.06	-0.42	0.30
Victivallaceae	0.42	-0.00	0.85	0.68	0.34	1.02	0.93	0.54	1.32

The “-” notation indicates the bacteria undetected in that country

We compared ENIGMA to the Wilcoxon rank sum test, a classical methods for identifying bacteria related with an environmental factor of interest [16]. Table 6 shows the bacteria significantly related to PD with *p*-value <0.05 in more than two countries. We observed that the bacteria detected by the Wilcoxon test were mostly included in those of ENIGMA (Table 5). Notably, all of the corrected *p*-values in Table 6 are larger than 0.05. This result shows that ENIGMA was superior to the Wilcoxon rank sum test in terms of identifying a larger number of associations between microbiota and PD.

**Table 6:** *p*-Value of Wilcoxon test

	Finland	Germany	USA
Lachnospiraceae	0.009371	0.719014	0.002839
Lactobacillaceae	0.030404	0.077771	0.000002
Pasteurellaceae	0.006493	0.495315	0.004232
Prevotellaceae	0.001303	0.030892	0.194592

**Fig. 9:**
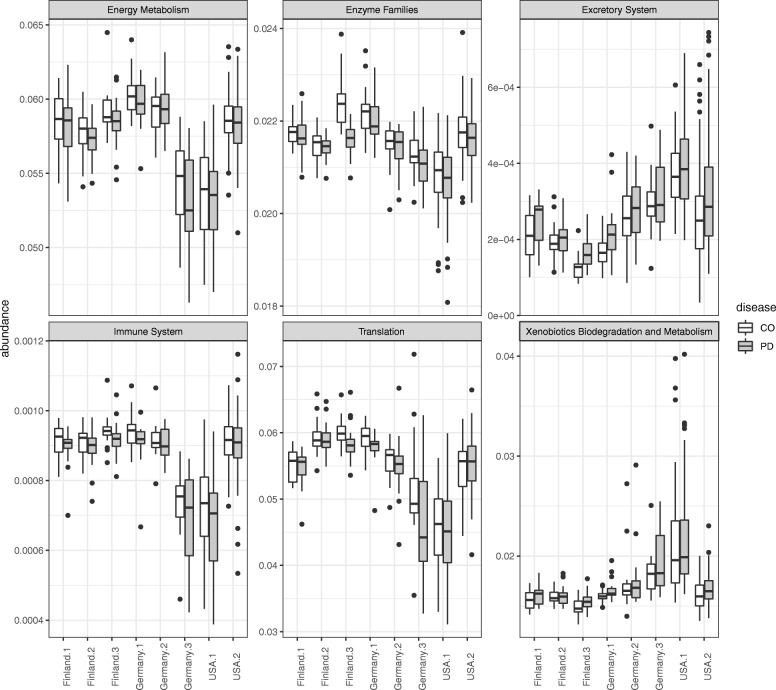
Predicted functional trait abundance in each country and cluster

Finally, we combined the results of ENIGMA to those of PICRUSt (version 1.1.3) [18] in order to evaluate which functions are related to PD. In the present study, PICRUSt was performed using the default settings. The Fig. 9 shows the functions exhibiting an increase and decrease from the median, which matched in all countries and clusters with respect to PD and control (CO). This result indicates that ENIGMA is a valuable tool for discovering new disease-related functions.

The analyses using real-world data thus show that ENIGMA can identify enterotype-like clusters and the associations between the gut microbiota and PD. Some of the results were strongly supported by those of previous studies.

## Conclusion

We proposed a novel hierarchical Bayesian model, ENIGMA, for discovering the underlying microbial community structures and associations between microbiota and their environmental factors from microbial metagenome data. ENIGMA is based on a probabilistic model of a microbial community structures and supplied with labels for one or more environmental factors of interest for each sample. The structures of each sample are modeled by a multinomial distribution whose parameters are represented independently by group and environmental effects of each sample, which prevents mixing of individual differences and the effects of interest. This framework enables the model to simultaneously learn (*i*) how microbes contribute to an underlying community structures (cluster) and (*ii*) how microbial compositional patterns are explained by environmental factors of interest. The effectiveness of ENIGMA was evaluated through experiments involving both synthetic and read-world datasets. These newly discovered clusters and associations estimated using ENIGMA can provide insight into the the mechanisms of a microbial communities.

The major limitation of ENIGMA is its scalability and efficiency, as the number of the parameters in the model increase proportionally with the number of taxa when the number of environmental factors of interest is large. Further studies should focus on developing a scalable probabilistic model of microbial compositions to analyze underlying microbial structures with a large number of these effects by using sparse parameter estimation [19]. We are also interested in developing a dynamic probabilistic model similar to that reported by Blei and Lafferty [20] for analyzing time-varying bacteria compositions during disease progression.

## Abbreviations

CH: Calinski-Harabasz
CO: Control
CP: Coverage probability
JSD: Jensen-Shannon divergence
MAP: Maximum a posteriori
OTU: Operational taxonomic units
PD: Parkinson’s disease
RMSE: Root mean squared error
SE: Standard error
